# Potential impact of the joint association of total bilirubin and gamma-glutamyltransferase with metabolic syndrome

**DOI:** 10.1186/s13098-019-0408-z

**Published:** 2019-02-04

**Authors:** Makoto Shiraishi, Muhei Tanaka, Hiroshi Okada, Yoshitaka Hashimoto, Shinichi Nakagawa, Muneaki Kumagai, Teruyuki Yamamoto, Hiromi Nishimura, Yohei Oda, Michiaki Fukui

**Affiliations:** 1 Department of Endocrinology and Metabolism, Graduate School of Medical Science, Kyoto Prefectural University of Medicine, 465 Kajii-cho, Kawaramachi-Hirokoji, Kamigyo-ku, Kyoto, 602-8566 Japan; 2 Department of Internal Medicine, Matsushita Memorial Hospital, Osaka, Japan; 3 Medical Corporation Soukenkai, Nishimura Clinic, Kyoto, Japan

**Keywords:** Bilirubin, Gamma-glutamyltransferase, Metabolic syndrome

## Abstract

**Background:**

Metabolic syndrome is characterized by the clustering of different metabolic abnormalities. Total bilirubin and gamma-glutamyltransferase (GGT) levels have been reported to be associated with this condition. However, the extent to which the interaction between these parameters affects metabolic syndrome is unknown. Therefore, we examined the association of total bilirubin and GGT levels with metabolic syndrome, and investigated the combined effect of the two parameters.

**Methods:**

In this retrospective cohort study, we analyzed 8992 middle-aged Japanese subjects (4586 men, 4406 women; mean age, 44.8 ± 9.3 years) without metabolic syndrome from a cohort of employees undergoing annual health examinations. They were divided into four groups according to median total bilirubin and GGT levels: both-low, GGT-high, total bilirubin-high, and both-high. The incident of metabolic syndrome was evaluated during a follow-up of 2.8 ± 1.2 years.

**Results:**

The incident rate of metabolic syndrome during the follow-up was 4.6% in the both-low group, 12.1% in the GGT-high group, 2.7% in the total bilirubin-high group, and 10.6% in the both-high group. Total bilirubin and GGT have an interaction effect on the risk of incident metabolic syndrome (*p *= 0.0222). The both-low [hazard ratio (HR), 1.37; 95% confidence interval (CI) 1.002–1.89], GGT-high (HR, 1.88; 95% CI 1.42–2.52), and both-high (HR, 2.07; 95% CI 1.56–2.80) groups showed an increased adjusted HR for incident metabolic syndrome after adjusting for covariates compared with the total bilirubin-high group.

**Conclusions:**

The simultaneous presence of high total bilirubin and low GGT levels may be associated with a lower incidence of metabolic syndrome.

## Background

Metabolic syndrome is a clustering of metabolic abnormalities including obesity, hyperglycemia, hypertriglyceridemia, hypertension, and decreased high-density lipoprotein (HDL) cholesterol [1, 2]. Besides the initially identified features of metabolic syndrome, several additional factors have been implicated in the underlying pathogenesis. These include chronic inflammation [3, 4], oxidative stress [5, 6], insulin resistance [6], hepatic steatosis [7], and adipokine level [8, 9]. Metabolic syndrome has been associated with many diseases such as type 2 diabetes [10], cardiovascular diseases [11], and fatty liver disease [12].

Recent studies have shown that total bilirubin [13–19] and gamma-glutamyltransferase (GGT) [20, 21] are closely associated with metabolic syndrome. Concerning total bilirubin, although previous cross-sectional studies [15, 17] and retrospective longitudinal studies [18, 19] showed an inverse association between total bilirubin and metabolic syndrome, Oda and Aizawa reported in 2013 that total bilirubin was not a risk factor for metabolic syndrome in Japanese men and women [18]. With regard to GGT, several cross-sectional studies [20, 21] found that a high level of GGT was positively associated with metabolic syndrome. Furthermore, Tao et al. concluded that GGT is a sensitive but moderately specific marker for the early diagnosis of metabolic syndrome in adults in Beijing, China [21]. In their cross-sectional study, Wang et al. reported that high total bilirubin levels had a protective effect against metabolic syndrome, whereas high GGT levels were risk factors for metabolic syndrome [22]. However, the extent to which the interaction between total bilirubin and GGT affects metabolic syndrome is unknown. Therefore, in the present study, we examined the relationship between total bilirubin and GGT and metabolic syndrome in middle-aged Japanese subjects. We also investigated the combined effect of total bilirubin and GGT on metabolic syndrome.

## Methods

### Subjects and study design

**Fig. 1:**
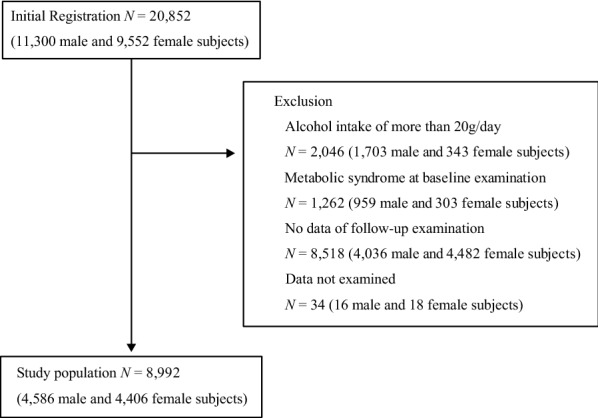
Flowchart of the inclusion and exclusion of subjects

The Nishimura Health Survey is an ongoing cohort investigation of risk factors for chronic diseases including hypertension, metabolic syndrome, diabetes mellitus, and chronic kidney disease. The Nishimura Clinic (Kyoto, Japan) provides regular health check-up for employees of various companies. In Japan, annual routine health examination of employees is legally mandated, and the employers usually pay all or most of the health-check costs. We performed a retrospective cohort study to assess the relationship between total bilirubin and GGT levels at baseline and incident metabolic syndrome during a follow-up of 2.8 ± 1.2 years. Among 20,852 subjects who underwent health examinations from April 1, 2013, to March 31, 2018, a total of 12,334 subjects underwent two or more health examinations. We excluded 34 subjects with data not examined on at least one variable. From the remaining 12,300 subjects, we excluded 2046 subjects with alcohol intake of > 20 g/day and 1262 subjects with metabolic syndrome at baseline. Finally, 8992 subjects were selected as eligible for the study (Fig. 1). The subjects were divided into four study groups according to the median values of total bilirubin and GGT: (i) both total bilirubin and GGT low (total bilirubin and GGT less than the median value, both-low group), (ii) total bilirubin low and GGT high (total bilirubin less than the median value and GGT equal to or higher than the median value, GGT-high group), (iii) total bilirubin high and GGT low (total bilirubin equal to or higher than the median value and GGT less than the median value, total bilirubin-high group), and (iv) both total bilirubin and GGT high (total bilirubin and GGT equal to or higher than the median value, both-high group).

In addition, all the subjects were divided into another four study groups according to the reference ranges of total bilirubin and GGT: (i) both total bilirubin and GGT levels within the reference range (total bilirubin and GGT levels equal to or lower than the upper reference range, both-reference group), (ii) total bilirubin level within the reference range and GGT level higher than the reference range (total bilirubin level equal to or lower than the upper reference range and GGT level higher than the upper reference range, GGT-higher than reference group), (iii) total bilirubin level above the reference range and GGT level within the reference range (total bilirubin level higher than the upper reference range and GGT level equal to or lower than the upper reference range, total bilirubin-higher than reference group), and (iv) both total bilirubin and GGT levels above the reference range (total bilirubin and GGT levels higher than the upper reference range, both-higher than reference group.

All procedures of the present study were approved by the local research ethics committee and were conducted in accordance with the Declaration of Helsinki. Informed consent was obtained from all subjects.

### Data collection and measurements

All subjects provided details of their demographics. Smoking was defined as current tobacco use. Alcohol drinking habits were evaluated by asking the subjects about the amount and frequency of intake of alcoholic beverages per week during the past month, and then estimating the mean ethanol intake per week. When subjects performed any kind of sports at least 30 min/day regularly, they were categorized as regular exercisers. Body mass index was calculated as weight in kilograms divided by height in meters squared. After an overnight fast, venous blood was collected for the measurement of the levels of various factors, including fasting plasma glucose, HDL cholesterol, triglycerides, total bilirubin, and GGT. We calculated Bil/GGT as the ratio of the total bilirubin value divided by the gamma-glutamyltransferas value. The reference ranges for total bilirubin and GGT were 1.7–20.5 μmol/L and 0.01–0.83 μkat/L, respectively.

### Prevalence of fatty liver disease

Abdominal ultrasonography, which was performed by trained technicians, was used for diagnosing fatty liver. Liver brightness and liver contrast were used for diagnosing fatty liver.

### Definition of metabolic syndrome

The diagnosis of metabolic syndrome was determined according to the joint interim statement of the International Diabetes Federation Task Force on Epidemiology and Prevention; the National Heart, Lung, and Blood Institute; the American Heart Association; the World Heart Federation; the International Atherosclerosis Society; and the International Association for the Study of Obesity, using the criteria for Asians [23]. Metabolic syndrome was diagnosed in the subjects when three or more of the following criteria were present: elevated blood pressure (systolic blood pressure ≥ 130 mmHg and/or diastolic blood pressure ≥ 85 mmHg and/or medication for hypertension, in both sexes), hyperglycemia (fasting plasma glucose ≥ 5.6 mmol/L and/or medication for diabetes, in both sexes), hypertriglyceridemia (serum triglycerides ≥ 1.70 mmol/L, in both sexes), low HDL cholesterol levels (serum HDL cholesterol < 1.03 mmol/L and/or medication for dyslipidemia in men and < 1.29 mmol/L and/or medication for dyslipidemia in women), and abdominal obesity (waist circumference ≥ 90 cm in men and ≥ 80 cm in women).

### Statistical analysis

Continuous variables are presented as mean ± 1 standard deviation and categorical variables as number (percentage). Differences in categorical and continuous variables across the four study groups were assessed using a Chi-square analysis and one-way analysis of variance, respectively. The hazard ratios (HRs) of the four study groups or Bil/GGT ratio for incident metabolic syndrome were calculated using univariate and multiple Cox regression analyses. The following variables were analyzed as potential covariates: age, body mass index, exercise and smoking status, and number of metabolic syndrome factors. We also tested for a potential interaction effect of the subgroups of total bilirubin and GGT levels on incident metabolic syndrome. To evaluate the predictive performance of the Bil/GGT ratio, we employed the time-dependent receiver operating characteristic (ROC) curve for censored survival data and the area under the ROC curve (AUC) as criteria. ROC analysis is a standard technique for assessing the performance of a continuous variable for binary classification. As the event occurrence is time-dependent, time-dependent ROC curves are more appropriate than conventional ones in our study. A *p*-value of < 0.05 was considered statistically significant. The level of significance for the interaction term was *p *< 0.1. Statistical analyses were performed using the JMP version 11.0 software (SAS Institute Inc., Cary, NC, USA). We also used R software version 3.4.1 and the “survival ROC” package to do the time-dependent ROC curve analysis.

## Results

Overall, the mean age of the subjects was 44.8 years (standard deviation, 9.3 years; range, 21–84 years), and 49.0% were women. The median total bilirubin levels in both men and women, men, and women were 13.7 μmol/L (interquartile range, 10.3–17.1 μmol/L), 15.4 μmol/L (12.0–18.8 μmol/L), and 12.0 μmol/L (10.3–15.4 μmol/L), respectively. The median GGT levels in both men and women, men, and women were 0.33 μkat/L (interquartile range, 0.23–0.52 μkat/L), 0.44 μkat/L (0.32–0.68 μkat/L), and 0.26 μkat/L (0.20–0.35 μkat/L), respectively. The characteristics of the subjects in each group (both-low, GGT-high, total bilirubin-high, and both-high) are shown in Table 1. In men, the incident rate of metabolic syndrome during the follow-up was 6.1% (79 of 1303) in the both-low group, 15.0% (199 of 1331) in the GGT-high group, 4.2% (42 of 1007) in the total bilirubin-high group, and 14.8% (140 of 945) in the both-high group. In women, the incident rate of metabolic syndrome during the follow-up was 3.0% (36 of 1186) in the both-low group, 8.5% (87 of 1026) in the GGT-high group, 1.4% (16 of 1125) in the total bilirubin-high group, and 6.8% (73 of 1069) in the both-high group. The prevalence of fatty liver disease in men was 19.3% (252 of 1303) in the both-low group, 42.8% (569 of 1331) in the GGT-high group, 16.2% (163 of 1007) in the total bilirubin-high group, and 42.1% (398 of 945) in the both-high group. In women, the prevalence of fatty liver disease was 5.6% (66 of 1186) in the both-low group, 17.5% (180 of 1026) in the GGT-high group, 4.2% (47 of 1125) in the total bilirubin-high group, and 10.4% (111 of 1069) in the both-high group. In all subjects, the main effect of high total bilirubin (*p *= 0.0001), the main effect of high GGT (*p *< 0.0001), and the interaction between total bilirubin and GGT (*p *= 0.0222) were all significant (Table 2). Cox regression analyses were performed to investigate the association between the four study groups and incident metabolic syndrome (Table 3). In all subjects, the both-low group [HR, 1.74; 95% confidence interval (CI) 1.28–2.40], GGT-high group (HR, 4.55; 95% CI 3.46–6.09), and both-high group (HR, 3.97; 95% CI 2.99–5.35) showed an increased unadjusted HR for incident metabolic syndrome compared with the total bilirubin-high group. In all subjects or in women, the both-low, GGT-high, and both-high groups showed an increased adjusted HR for incident metabolic syndrome after adjusting for covariates compared with the total bilirubin-high group. On the other hand, in male subjects, the GGT-high and both-high groups showed an increased adjusted HR for incident metabolic syndrome after adjusting for covariates compared with the total bilirubin-high group; however, the risk in the both-low group did not differ from that of the total bilirubin-high group.

**Table 1:** Clinical characteristics of the study participants according to total bilirubin and gamma-glutamyltransferase classifications

(A) Men	Total bilirubin (μmol/L)				*p*-value
	< 15.4		≥ 15.4		
	Gamma-glutamyltransferase (μkat/L)		Gamma-glutamyltransferase (μkat/L)		
	< 0.44	≥ 0.44	< 0.44	≥ 0.44	
*n*	1303	1331	1007	945	
Age (years)	44.3 ± 10.0	45.7 ± 9.4	43.0 ± 9.6	45.3 ± 9.2	< 0.0001
Follow-up interval (years)	2.8 ± 1.2	2.8 ± 1.2	2.8 ± 1.2	2.8 ± 1.2	0.5846
Body mass index (kg/m^2^)	22.5 ± 2.5	23.9 ± 3.0	22.1 ± 2.4	23.7 ± 3.0	< 0.0001
Current smoking	352 (27.0)	385 (28.9)	150 (14.9)	146 (15.5)	< 0.0001
Exercise habit	315 (24.2)	293 (22.0)	242 (24.0)	218 (23.1)	0.5476
Waist circumference (cm)	81.0 ± 7.1	84.8 ± 7.6	79.7 ± 6.9	84.3 ± 8.0	< 0.0001
Waist circumference ≥ 90 cm	139 (10.7)	276 (20.7)	80 (7.9)	188 (19.9)	< 0.0001
Systolic blood pressure (mmHg)	117.9 ± 16.1	121.3 ± 14.8	117.8 ± 14.0	121.2 ± 14.6	< 0.0001
Diastolic blood pressure (mmHg)	72.2 ± 11.8	75.0 ± 11.4	72.0 ± 10.6	75.4 ± 11.2	< 0.0001
Systolic blood pressure ≥ 130 mmHg and/or diastolic blood pressure ≥ 85 mmHg and/or medication for hypertension	294 (22.6)	395 (29.7)	227 (22.5)	281 (29.7)	< 0.0001
Fasting plasma glucose (mmol/L)	5.3 ± 0.6	5.5 ± 0.7	5.3 ± 0.5	5.5 ± 0.8	< 0.0001
Fasting plasma glucose ≥ 5.6 mmol/L and/or medication for diabetes	63 (4.8)	106 (8.0)	37 (3.7)	64 (6.8)	< 0.0001
HDL cholesterol (mmol/L)	1.6 ± 0.4	1.6 ± 0.4	1.7 ± 0.4	1.6 ± 0.4	< 0.0001
HDL cholesterol < 1.03 mmol/L and/or medication for dyslipidemia	74 (5.7)	102 (7.7)	41 (4.1)	67 (7.1)	0.0021
Triglycerides (mmol/L)^a^	1.1 ± 0.6	1.4 ± 0.9	1.0 ± 0.5	1.3 ± 0.7	< 0.0001
Triglycerides ≥ 1.7 mmol/L	124 (9.5)	341 (25.6)	68 (6.8)	199 (21.1)	< 0.0001
Number of metabolic syndrome factors	0.5 ± 0.7	0.9 ± 0.8	0.4 ± 0.7	0.8 ± 0.8	< 0.0001
Prevalence of fatty liver disease	252 (19.3)	569 (42.8)	163 (16.2)	398 (42.1)	< 0.0001
Total bilirubin (μmol/L)	12.0 ± 2.6	12.1 ± 2.6	22.4 ± 6.7	22.4 ± 7.1	< 0.0001
Gamma-glutamyltransferase (μkat/L)	0.3 ± 0.1	0.9 ± 0.6	0.3 ± 0.1	0.9 ± 0.8	< 0.0001
Bil/GGT ratio	39.5 ± 13.1	17.1 ± 7.4	74.6 ± 28.7	31.8 ± 16.8	< 0.0001
Incident metabolic syndrome during follow-up	79 (6.1)	199 (15.0)	42 (4.2)	140 (14.8)	< 0.0001

(B) Women	Total bilirubin (μmol/L)				*p*-value
	< 12.0		≥ 12.0		
	Gamma-glutamyltransferase (μkat/L)		Gamma-glutamyltransferase (μkat/L)		
	< 0.26	≥ 0.26	< 0.26	≥ 0.26	
*n*	1186	1026	1125	1069	
Age (years)	43.3 ± 8.5	46.9 ± 9.1	43.3 ± 8.6	46.7 ± 9.2	< 0.0001
Follow-up interval (years)	2.8 ± 1.2	2.8 ± 1.2	2.9 ± 1.3	2.8 ± 1.2	0.2852
Body mass index (kg/m^2^)	20.8 ± 2.6	21.4 ± 3.3	20.2 ± 2.3	20.7 ± 3.2	< 0.0001
Current smoking	65 (5.5)	89 (8.7)	30 (2.7)	34 (3.2)	< 0.0001
Exercise habit	176 (14.8)	175 (17.1)	191 (17.0)	217 (20.3)	0.0078
Waist circumference (cm)	75.2 ± 7.1	77.3 ± 8.6	74.1 ± 6.6	75.4 ± 8.1	< 0.0001
Waist circumference ≥ 80 cm	287 (24.2)	351 (34.2)	204 (18.1)	280 (26.2)	< 0.0001
Systolic blood pressure (mmHg)	109.9 ± 15.1	114.6 ± 18.0	110.2 ± 15.1	114.5 ± 17.5	< 0.0001
Diastolic blood pressure (mmHg)	65.8 ± 10.3	69.8 ± 12.3	66.1 ± 10.5	69.4 ± 12.1	< 0.0001
Systolic blood pressure ≥ 130 mmHg and/or diastolic blood pressure ≥ 85 mmHg and/or medication for hypertension	116 (9.8)	204 (19.9)	124 (11.0)	201 (18.8)	< 0.0001
Fasting plasma glucose (mmol/L)	5.0 ± 0.5	5.1 ± 0.4	5.0 ± 0.4	5.1 ± 0.5	< 0.0001
Fasting plasma glucose ≥ 5.6 mmol/L and/or medication for diabetes	15 (1.3)	28 (2.7)	9 (0.8)	28 (2.6)	0.0006
HDL cholesterol (mmol/L)	2.0 ± 0.4	2.0 ± 0.4	2.1 ± 0.4	2.1 ± 0.5	< 0.0001
HDL cholesterol < 1.29 mmol/L and/or medication for dyslipidemia	52 (4.4)	70 (6.8)	31 (2.8)	68 (6.4)	< 0.0001
Triglycerides (mmol/L)^a^	0.8 ± 0.4	0.9 ± 0.4	0.7 ± 0.3	0.8 ± 0.4	< 0.0001
Triglycerides ≥ 1.7 mmol/L	37 (3.1)	47 (4.6)	15 (1.3)	34 (3.2)	0.0002
Number of metabolic syndrome factors	0.4 ± 0.6	0.7 ± 0.8	0.3 ± 0.6	0.6 ± 0.7	< 0.0001
Prevalence of fatty liver disease	66 (5.6)	180 (17.5)	47 (4.2)	111 (10.4)	< 0.0001
Total bilirubin (μmol/L)	9.9 ± 1.9	10.0 ± 1.8	17.6 ± 4.8	17.8 ± 5.3	< 0.0001
Gamma-glutamyltransferase (μkat/L)	0.2 ± 0.03	0.5 ± 0.3	0.2 ± 0.03	0.5 ± 0.4	< 0.0001
Bil/GGT ratio	50.6 ± 13.6	26.7 ± 10.1	91.0 ± 32.7	47.3 ± 20.2	< 0.0001
Incident metabolic syndrome during follow-up	36 (3.0)	87 (8.5)	16 (1.4)	73 (6.8)	< 0.0001

Continuous variables are presented as mean ± 1 standard deviation and categorical variables are presented as number (percentage). Differences in categorical and continuous variables across the four study groups were assessed using Chi-square analysis and one-way analysis of variance, respectively

*HDL* high-density lipoprotein, *Bil/GGT ratio* total bilirubin to gamma-glutamyltransferase ratio

^a^Values were analyzed after log transformation

**Table 2:** Cox regression analysis of significant main effects and interactions of total bilirubin and gamma-glutamyltransferase on incident metabolic syndrome

Predictor variables	Men and women		Men		Women	
	Hazard ratio (95% CI)	*p*-value	Hazard ratio (95% CI)	*p*-value	Hazard ratio (95% CI)	*p*-value
Main effects						
Total bilirubin (low total bilirubin)						
High total bilirubin	0.70 (0.59–0.85)	0.0001	0.82 (0.66–1.01)	0.0677	0.60 (0.43–0.84)	0.0022
Gamma-glutamyltransferase (low gamma-glutamyltransferase)						
High gamma-glutamyltransferase	3.21 (2.69–3.87)	< 0.0001	2.99 (2.42–3.73)	< 0.0001	3.67 (2.66–5.20)	< 0.0001
Interaction effects						
Total bilirubin × gamma-glutamyltransferase		0.0222		0.0764		0.0857

The overall total bilirubin range was divided into two subranges: low total bilirubin (< 15.4 μmol/L in men and < 12.0 μmol/L in women) and high total bilirubin (≥ 15.4 μmol/L in men and ≥ 12.0 μmol/L in women). The overall gamma-glutamyltransferase range was also divided into two subranges: low gamma-glutamyltransferase (< 0.44 μkat/L in men and < 0.26 μkat/L in women) and high gamma-glutamyltransferase (≥ 0.44 μkat/L in men and ≥ 0.26 μkat/L in women). The level of significance for the interaction term was *p *< 0.1

*CI* confidence interval

**Table 3:** Hazard ratios and 95% confidence intervals for incident metabolic syndrome according to classifications of total bilirubin and gamma-glutamyltransferase

	Unadjusted	Model 1	Model 2
(A) Men and women			
Total bilirubin high and GGT low	1.00	1.00	1.00
Both total bilirubin and GGT low	1.74 (1.28–2.40)	1.38 (1.01–1.91)	1.37 (1.002–1.89)
Total bilirubin low and GGT high	4.55 (3.46–6.09)	2.45 (1.86–3.30)	1.88 (1.42–2.52)
Both total bilirubin and GGT high	3.97 (2.99–5.35)	2.49 (1.87–3.37)	2.07 (1.56–2.80)
(B) Men			
Total bilirubin high and GGT low	1.00	1.00	1.00
Both total bilirubin and GGT low	1.48 (1.03–2.17)	1.22 (0.85–1.80)	1.21 (0.83–1.77)
Total bilirubin low and GGT high	3.65 (2.64–5.16)	2.17 (1.56–3.08)	1.69 (1.22–2.39)
Both total bilirubin and GGT high	3.63 (2.60–5.19)	2.25 (1.60–3.23)	1.86 (1.32–2.67)
(C) Women			
Total bilirubin high and GGT low	1.00	1.00	1.00
Both total bilirubin and GGT low	2.21 (1.25–4.09)	1.86 (1.05–3.44)	1.78 (1.005–3.30)
Total bilirubin low and GGT high	6.09 (3.68–10.76)	3.14 (1.88–5.59)	2.36 (1.41–4.19)
Both total bilirubin and GGT high	4.89 (2.93–8.71)	3.01 (1.79–5.38)	2.56 (1.52–4.56)

Model 1: adjusted for age, body mass index, exercise, and smoking status. Model 2: adjusted for model 1 and the number of metabolic syndrome factors. The overall total bilirubin range was divided into two subranges: low total bilirubin (< 15.4 μmol/L in men and < 12.0 μmol/L in women) and high total bilirubin (≥ 15.4 μmol/L in men and ≥ 12.0 μmol/L in women). The overall gamma-glutamyltransferase range was also divided into two subranges: low gamma-glutamyltransferase (< 0.44 μkat/L in men and < 0.26 μkat/L in women) and high gamma-glutamyltransferase (≥ 0.44 μkat/L in men and ≥ 0.26 μkat/L in women)

Three thousand five hundred eleven (76.6%) men and 3895 (88.4%) women had the reference range of bilirubin level, and 3785 (82.5%) men and 4251 (96.5%) women had the reference range of GGT level. There were 2896 men and 3772 women in both-reference group, 615 and 123 in GGT-higher than reference group, 889 and 479 in total bilirubin-higher than reference group, and 186 and 32 in both-higher than reference group. In all subjects, the GGT-higher than reference group (HR, 3.86; 95% CI 2.93–5.14), and both-higher than reference group (HR, 3.58; 95% CI 2.40–5.25) showed an increased unadjusted HR for incident metabolic syndrome compared with the total bilirubin-higher than reference group.

In all subjects or in men or women, the Bil/GGT ratio showed a decreased adjusted HR for incident metabolic syndrome after adjusting for covariates (Table 4). To evaluate the performance of the Bil/GGT ratio for predicting metabolic syndrome, time-dependent ROC curve analysis was performed. This analysis provided the AUC for each follow-up time. The AUC and optimized cut-off value for the Bil/GGT ratio to differentiate incident metabolic syndrome at 3 years were 0.708 and 41.0 in all subjects, 0.671 and 28.0 in men, and 0.713 and 41.9 in women. The AUC and optimized cut-off value at 5 years were 0.693 and 41.0 in all subjects, 0.662 and 34.8 in men, and 0.688 and 41.9 in women.

**Table 4:** Hazard ratios and 95% confidence intervals of total bilirubin to gamma-glutamyltransferase ratio for incident metabolic syndrome

	Unadjusted	Model 1	Model 2
(A) Men and women			
Total bilirubin to gamma-glutamyltransferase ratio, per 10.0 increment	0.73 (0.70–0.76)	0.84 (0.80–0.87)	0.89 (0.85–0.92)
(B) Men			
Total bilirubin to gamma-glutamyltransferase ratio, per 10.0 increment	0.76 (0.72–0.80)	0.83 (0.79–0.88)	0.89 (0.84–0.94)
(C) Women			
Total bilirubin to gamma-glutamyltransferase ratio, per 10.0 increment	0.74 (0.69–0.79)	0.85 (0.79–0.90)	0.90 (0.84–0.96)

Model 1: adjusted for age, body mass index, exercise, and smoking status. Model 2: adjusted for model 1 and the number of metabolic syndrome factors

## Discussion

Our study has four main findings. First, both total bilirubin and GGT were identified as important predictors of metabolic syndrome in middle-aged Japanese subjects without a daily alcohol intake of > 20 g/day. Second, these findings persisted even after adjustment for several factors in all subjects or in women. Third, we observed an interaction effect between total bilirubin and GGT on the risk of incident metabolic syndrome. Finally, we demonstrated that the Bil/GGT ratio was an important predictor of metabolic syndrome. Besides, we were able to provisionally suggest optimal cut-off values for the Bil/GGT ratio to predict metabolic syndrome. Taken together, these findings suggest that total bilirubin and GGT are independently associated with incident metabolic syndrome. In other words, the simultaneous presence of high total bilirubin and low GGT level in a subject was associated with a lower risk of metabolic syndrome.

Recent studies have revealed that total bilirubin is closely related to metabolic syndrome [13–20]. Some of the studies had a cross-sectional design [15, 17] and others were retrospective longitudinal studies [18, 19]. Most of them showed an inverse association between total bilirubin and metabolic syndrome. On the other hand, Oda and Aizawa reported in 2013 that total bilirubin was not a risk factor for metabolic syndrome in Japanese men and women [18]. Oxidative stress has also been associated with metabolic syndrome [5, 6]. The rate-limiting step in heme degradation is catalyzed by heme oxygenase, which results in the release of equimolar quantities of ferrous ion, carbon monoxide, and biliverdin. This biliverdin is converted to bilirubin, the major physiological antioxidant, by biliverdin reductase [24]. Bilirubin is a potent antioxidant that can protect cells from a 10,000-fold excess of hydrogen peroxide [25]. The redox cycle of bilirubin mediated by biliverdin reductase contributes to the potent physiologic antioxidant actions of bilirubin [26]. Our study also found a negative relationship between total bilirubin and metabolic syndrome. Thus, elevated total bilirubin can be a protective factor against metabolic syndrome.

With regard to GGT, several reports suggested a highly significant relationship between GGT and metabolic syndrome [20, 21, 27–30]. Xu et al. reported a 4.37-fold increased risk of metabolic syndrome in the highest GGT quartiles after adjusting for age, sex, smoking, alcohol use, and body mass index in a longitudinal study with 5404 subjects [27]. Lee et al. reported a similar finding with an odds ratio of 2.97 in the highest GGT quartile after adjustment for age and drinking status in a cross-sectional study of 3508 subjects [28]. In a cross-sectional study of 7390 adults in Taiwan, Hwang et al. reported an odds ratio of 45.2 in the highest GGT quartile after adjusting for age, body mass index, history of alcoholic fatty liver disease, and medication use [29]. Oh et al. demonstrated an elevated GGT as a sensitive marker of metabolic syndrome in a 4-year cohort study of 3698 Korean male workers [30]. Furthermore, Tao et al. reported that GGT is a sensitive but moderately specific marker for the early diagnosis of metabolic syndrome in adults in Beijing, China [21]. Whitfield [31] and Stark [32] suggested that increases in serum GGT may initiate extracellular glutathione transport into the cells of organ systems, resulting in cellular oxidative stress. Nakanishi et al. supported the idea that a moderate increase of GGT may be a mediator of low-grade systemic inflammation, and explained the strong association of serum GGT with many cardiometabolic risk factors and diseases [33]. In the study by Lee et al. serum GGT level was a predictive factor of the future levels of inflammation and oxidative stress markers, such as fibrinogen, uric acid, C-reactive protein, and F2-isoprostanes [34]. In the present study, GGT was also found to be positively associated with metabolic syndrome. Therefore, GGT may be one of the predictive factors of metabolic syndrome.

A previous cross-sectional study demonstrated that GGT activity was weakly positively correlated with bilirubin levels, whereas bilirubin levels decreased progressively with the number of metabolic syndrome components as the mean GGT activity increased [35]. Our present findings are in line with the results of the previous study. In our study, GGT levels were positively associated with bilirubin levels in all subjects (r = 0.076, *p *< 0.0001) and in women (r = 0.044, *p *= 0.0035); however, no association was found between GGT and bilirubin levels in men (r = 0.005, *p *= 0.7216). Nonetheless, the simultaneous presence of high total bilirubin and low GGT level in a subject was associated with a lower risk of metabolic syndrome. As the convergence of the pro-oxidant potential of the association of higher GGT activity and lower bilirubin levels could contribute significantly to the increased systemic oxidative stress, this apparent contradiction may reflect its implication for the effect of the interaction between GGT and bilirubin levels.

Recently, fatty liver disease is considered as a strong determinant for the development of metabolic syndrome [21, 36, 37]. Interestingly, in our study, four study groups according to the median values of total bilirubin and GGT were significantly associated with prevalence of fatty liver disease. Therefore, these results support the concept that both total bilirubin and GGT were identified as important predictors of metabolic syndrome.

The present study has four limitations. First, we could not exclude subjects who reported a history of known liver disease, including viral, genetic, autoimmune, and drug-induced liver disease. Since the high levels of bilirubin and GGT may be clinically related to very serious liver diseases, we also tested for the HRs of another four study groups according to the reference ranges of total bilirubin and GGT for incident metabolic syndrome. The GGT-higher than reference group and both-higher than reference group showed an increased unadjusted HR for incident metabolic syndrome compared with the total bilirubin-higher than reference group. The result for study groups according to the reference ranges were almost in line with the result for study groups according to the median values. Second, we analyzed the data of subjects who visited the health promotion center as part of the mandatory annual health check-up for employees of various companies, and this group might not be representative of the general population. Third, because of funds shortage, we did not measure the parameters of oxidative stress, mediators of inflammation, and sex hormones in our study subjects. Fourth, male subjects in the GGT-high group and both-high group showed an increased adjusted HR for incident metabolic syndrome after adjusting for covariates compared with the total bilirubin-high group; however, the risk in the both-low group did not differ from that of the total bilirubin-high group. We cannot explain this sex difference, although the sex difference in the correlation coefficient between GGT and bilirubin levels may have resulted in the discrepancy.

## Conclusion

We observed an interaction effect between total bilirubin and GGT on the risk of incident metabolic syndrome. The simultaneous presence of high total bilirubin and low GGT levels may be favorable to the development of incident metabolic syndrome.

## Abbreviations

HDL: high-density lipoprotein
GGT: gamma-glutamyltransferase
HR: hazard ratio
ROC: receiver operating characteristic
AUC: area under the ROC curve
